# Comparison of Operative and Non-Operative Treatment of Acute Undisplaced or Minimally-Displaced Scaphoid Fractures: A Meta-Analysis of Randomized Controlled Trials

**DOI:** 10.1371/journal.pone.0125247

**Published:** 2015-05-05

**Authors:** Longxiang Shen, Jianfei Tang, Congfeng Luo, Xuetao Xie, Zhiquan An, Changqing Zhang

**Affiliations:** Department of Orthopedic Surgery, Shanghai Jiao Tong University Affiliated Sixth People’s Hospital, 600 Yishan Road, Shanghai, 200233, PR China; University of New Mexico Cancer Center, UNITED STATES

## Abstract

**Background:**

Traditionally, acute undisplaced or minimally-displaced scaphoid fractures are treated by casting in short- or long-arm casts. Although reports have shown that operative treatment is safe, effective and produces satisfactory results, outcomes from current studies comparing these two methods are questionable. The aim of this meta-analysis was to evaluate the effects of operative versus non-operative treatment for acute undisplaced or minimally-displaced scaphoid fractures in adults.

**Methods:**

Computerized searches were performed without language restrictions and all randomized controlled studies providing information on the effects of operative versus non-operative treatment on the outcomes of acute undisplaced or minimally-displaced scaphoid fractures were included. The weighted and standard mean difference (WMD and SMD) or the relative risk (RR) were calculated for continuous or dichotomous data respectively.

**Results:**

A total of six studies reported in seven publications were included, representing data on 340 fractures. Meta-analysis indicated that operative treatment resulted in significantly better functional outcomes in the short term when compared with non-operative treatment. Consistently, patients who accepted surgery had a more rapid return to work. Further, surgery was advantageous in preventing delayed union of the fractures, a finding supported by the results of analysis of the time to fracture union. A number-needed-to-treat analysis revealed that more than 20 patients would have to undergo operative treatment to prevent one delayed union.

**Conclusion:**

Acute undisplaced or minimally-displaced scaphoid fractures demonstrate faster recovery with operative treatment; however, the current meta-analysis does not provide evidence supporting the routine use of operative treatment for all acute undisplaced or minimally-displaced scaphoid fractures.

## Introduction

Traditionally, undisplaced, stable scaphoid fractures are treated by casting in short- or long-arm casts. Cast immobilization always involves prolonged immobilization of at least 12 weeks[1], but it has been demonstrated that union can be achieved in greater than 90% of affected individuals with this method[2]. However, prolonged immobilization disrupts collagen homeostasis resulting in loss of normal connective tissue characteristics, which normally allow tendons to glide and the joint capsule to stretch[3]. Clearly this management option can result in complications that may delay rehabilitation, as indicated by some studies in the literature that suggest poorer outcomes after prolonged immobilization[1,4]. In theory, early internal fixation has the benefits of early return of wrist movement, a higher rate of union, an early return to work and sport, and avoidance of the need for a plaster cast[3]. Although reports have shown that operative treatment is safe, effective and produces satisfactory results[5,6], the optimal management of undisplaced or minimally-displaced scaphoid fractures has been the focus of much debate[3,7].

Recently, a few randomized controlled trials (RCTs) regarding operative versus non-operative treatment in the management of acute undisplaced or minimally-displaced scaphoid fractures have been published. However, the relatively small sample size (n = 25–88) in each published study rendered the results inconclusive and controversial. Recently, a meta-analyses of RCTs compared the effectiveness of surgical versus non-surgical treatment of acute undisplaced or minimally-displaced scaphoid fractures[8]. Regrettably, a prospective controlled study[9], which was confirmed by its corresponding author, was non-randomized, but was included and analyzed in the meta-analysis[8]. Furthermore, sub-group analyses rather than independent analyses were used in the management of the data concerning complications, thereby making the conclusions questionable. Another pairwise and network meta-analysis of RCTs[10], which only included data of complications, range of motion and grip strength, produced conclusions which were not comprehensive. In order to make a more precise estimation, we performed a meta-analysis based on RCTs.

The aim of the current meta-analysis was to investigate the outcomes of operative treatment for minimally-displaced and undisplaced scaphoid fractures compared with non-operative treatment; furthermore, we also attempted to illuminate the limitations of current studies and to provide suggestions for further studies to evaluate these therapeutic options for the treatment of acute scaphoid fractures.

## Methods

### Search Strategy

We performed this meta-analysis following the guidelines of the PRISMA statement[11]. Computerized searches were performed without language restrictions on March 16, 2013 and an updated computerized search was performed on 31 December, 2014 using the phrase, “scaphoid fractures” limited with “randomized controlled trial” using PubMed (1949–2014), Web of Knowledge (1950–2014), BioMed Central (2000–2014), ScienceDirect (1995–2014) and EMBASE (1966–2014), as well as searching the Cochrane Central Register of Controlled Trials (CENTRAL) (1948–2014). Reference lists of review articles regarding the treatment of scaphoid fractures were scanned in order to find additional studies. Additionally, a manual search of English scientific literature was performed by cross-checking the bibliographies of all primary articles and previously published systematic reviews and meta-analyses. The inclusion criteria were: (a) randomized controlled studies on patients with acute undisplaced or minimally-displaced scaphoid fractures, (b) treatment compared operative versus non-operative methods. Exclusion criteria included: (a) non-randomized controlled trials, (b) trials focused on delayed union or nonunion of scaphoids, (c) pediatric fractures.

All identified studies were reviewed by all of the authors and information was carefully extracted independently by two reviewers (LS and JT); Any disagreements between the authors were resolved by discussion to reach a consensus. The quality of included RCTs was evaluated using the Jadad scale, with a score less than 3 being indicative of low quality[12]. The risk of bias of each eligible study was assessed in accordance with the Cochrane risk of bias tool[13].

### Statistical Analysis

From the selected articles, data extracted comprised: (a) the functional outcome, which was the primary outcome, measured using the Patient Evaluation Measure, a modified Green/O'Brien score and Patient-Related Wrist Evaluation (PRWE), (b) the range of motion (ROM), grip and pinch strength, (c) duration of absence from work and (d) final complications and time to fracture union. We sent an e-mail to each corresponding author of the included studies in an attempt to obtain the original data. In some instances standard deviations (SDs) were not presented; we calculated these from the *P* value or confidence interval (CI) using the method described in the Cochrane Handbook for Systematic Reviews of Interventions for conversion [13].

The data at the same time points of follow-up were pooled. For the meta-analysis of continuous variables using the same scales, the weighted mean difference (WMD) with a 95% CI was used, while the standardized mean difference (SMD) with a 95% CI was used for continuous variables using different scales, for example, function scores with different measurement scales. For dichotomous variables, the relative treatment effect was expressed as relative risk (RR) with a 95% CI. We anticipated the presence of clinical heterogeneity, based on the facts that fixation methods, surgical approach and cast immobilization varied among the RCTs. Because the test for heterogeneity had low statistical power, the presence of heterogeneity was assumed *a priori*, and the random effects model was used in all the analyses. A sensitivity analysis was performed by investigating the effect of each individual study on the pooled effect size. Funnel plots were used to assess possible publication bias. Because of the limited data available from each RCT for the pooled analysis, we did not perform subgroup analysis based on the treatment methods, for example, percutaneous or open screw fixation, short- or long-arm cast. A *P*-value less than 0.05 was considered statistically significant. Analyses were performed using the Stata/SE 10.0 program (Stata Corporation).

## Results

### Search Results and Characteristics of Selected Studies

**Fig 1:**
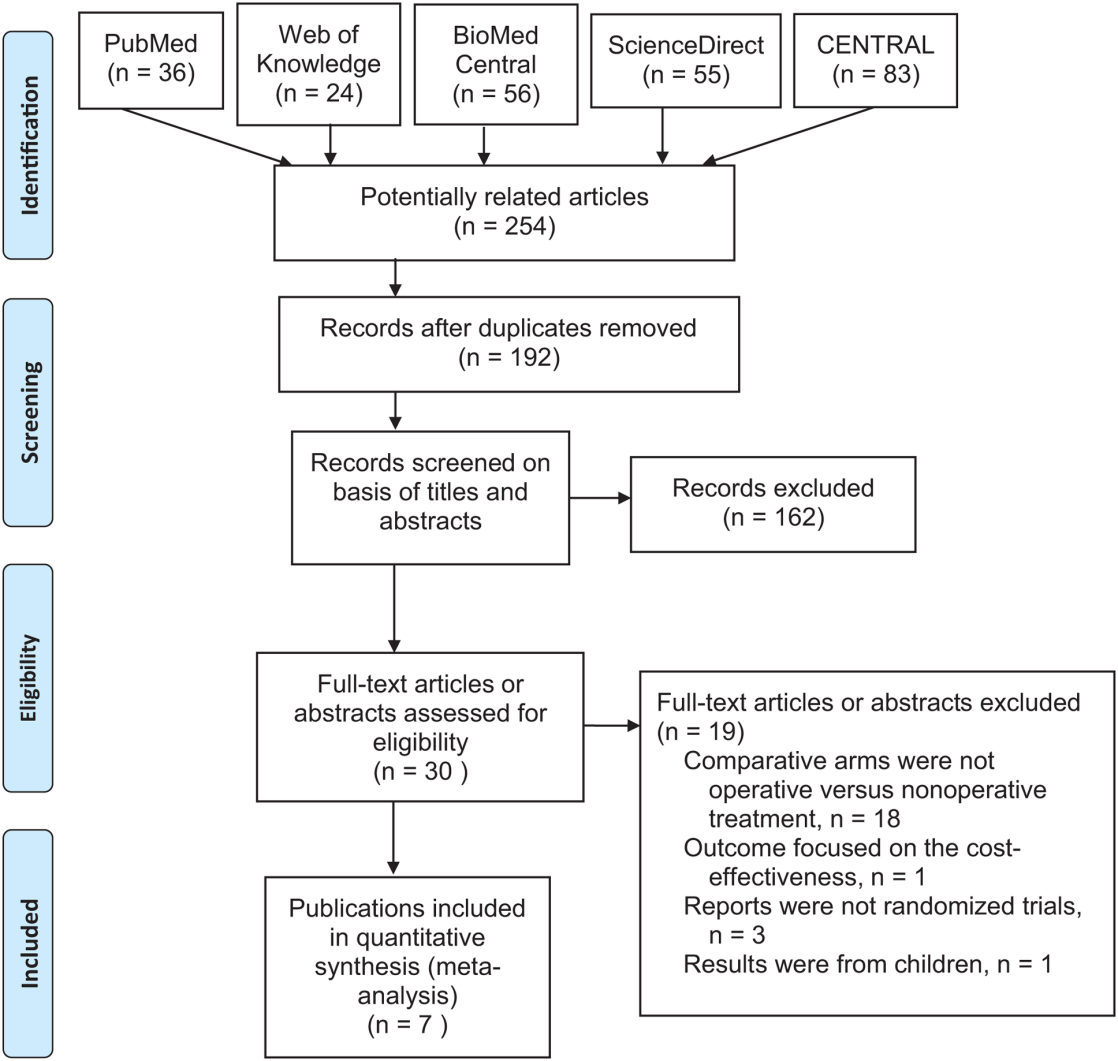
Flowchart of selection of studies and specific reasons for exclusion from the present meta-analysis. CENTRAL, Cochrane Central Register of Controlled Trials.

The analysis yielded a total of six articles eligible for analysis [14,15,16,17,18,19] (Fig 1 and Table 1). One report by Dias *et al*.[20] provided further follow-up data of their previous study [16]. The two publications were considered to form one study and were combined in the present analysis.

**Table 1:** Main characteristics of all eligible studies included in the analysis.

Author Year	Age (years)	Number of fractures (operative/nonoperative)	Female, n (operative/nonoperative)	Operative treatment	Nonoperative treatment	Follow-up time	Outcomes measured	Jadad score [12]
McQueen et al. [15] 2008	17–65	30/30	4/6	Percutaneous Acutrak screw fixation.	Colles cast with the thumb free.	52 weeks	ROM, the grip and pinch strength, the modified Green/O’Brien functional score, time of return to work and sports, union.	4
Vinnars et al. [14] 2008	17–65	26/26	-	Fractures were openly fixated with a Herbert screw.	Below-elbow scaphoid cast.	Mean 10.2 (8–13) years	DASH and the Patient Rated Wrist Evaluation scores, Complications, time to return duty, cost assessment. Radiographic assessments, ROM.	3
Dias et al. [20,16] 2008,2005	16–61	44/44	4/5	Fractures were openly fixated with a Herbert screw or a cannulated Whipple screw.	Below-the-elbow cast with the thumb left free.	12 and 93 months	Pain, ROM, grip strength, the Patient Evaluation Measure Questionnaire, complications, the date of return to work.	4
Adolfsson et al. [17] 2001	15–75	25/28	5/9	Percutaneous Acutrak screw fixation.	Below elbow plaster cast.	A minimum of 16 weeks	Union, complications, time to fracture union, ROM, grip strength.	2
Saedén et al. [19] 2001	15–>50	32/30	5/8	Fractures were openly fixated with a Herbert screw.	Short-arm cast.	10.2–12.8 years	Tenderness, ROM, strength, Union. The duration of sick leave.	2
Bond et al. [18] 2001	18–34	11/14	2/1	Percutaneous Acutrak screw fixation.	Long-arm thumb-spica cast.	Mean 25 (24–27) months	Union, grip strength, ROM, time to return duty, complications, patient’s final satisfaction	2

**Fig 2:**
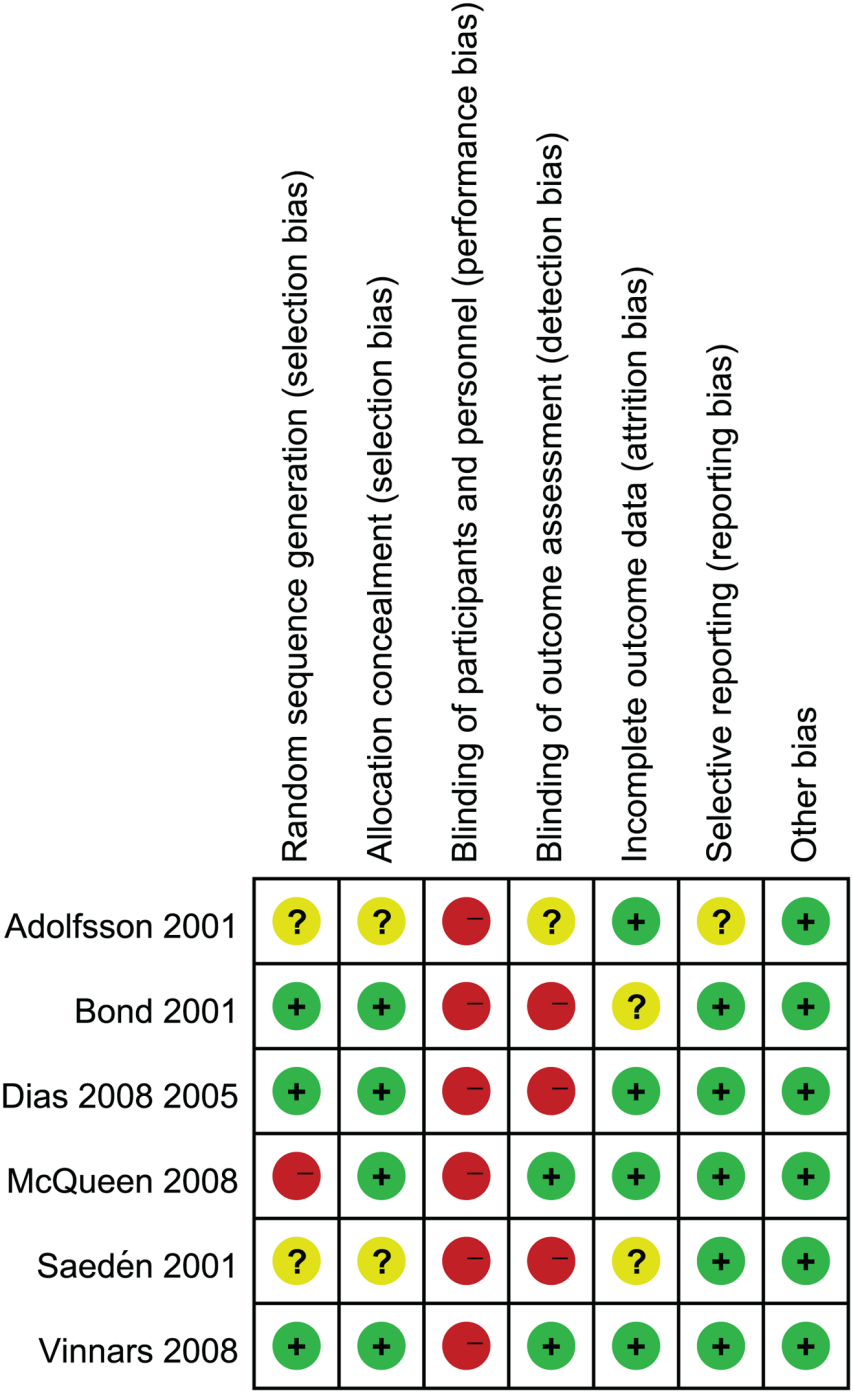
Risk of bias summary: review authors' judgements about each risk of bias item for each included study.

**Fig 3:**
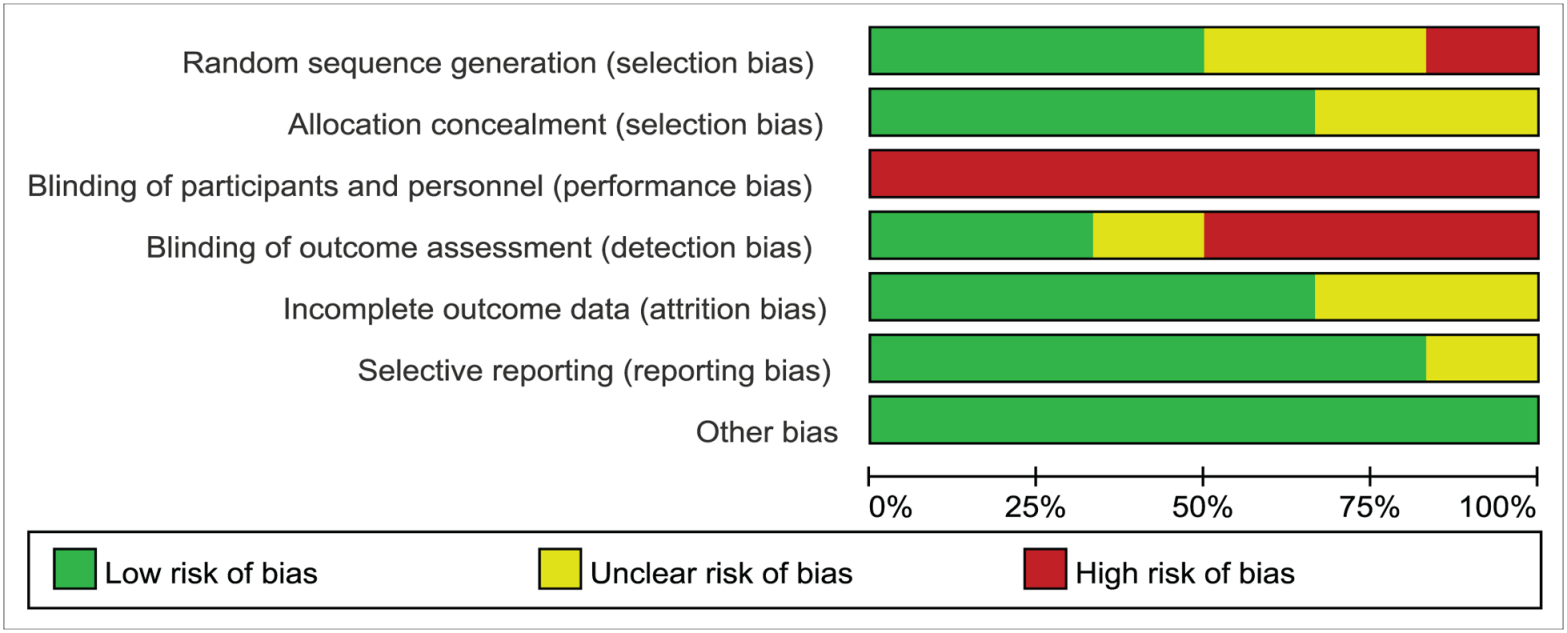
Risk of bias graph: review authors' judgements about each risk of bias item presented as percentages across all included studies.

The quality of each study was graded and scored from 2 to 4 according to the Jadad scoring system[12]. Sample sizes of the included studies were 25 to 88. In total, 340 fractures were included in our meta-analysis. Among them, 168 fractures were randomized to an operative treatment group, and 172 to a non-operative treatment group. Two studies[15,17] were performed in two centers, and the remainder were performed in a single center[14,16,18,19]. The risk of bias assessment of the included studies was presented in Fig 2. Judgments regarding each risk of bias item are presented as percentages across all the included studies in Fig 3. Three studies included allocation concealment[14,16,18] while the other three studies did not[15,17,19]. There were no double-blind studies. The range of follow-up time was 16 weeks to13 years in the primary studies. Two publications[15,16] reported outcome data at different times after treatment, while the remaining studies[14,17,18,19] reported outcome data at final follow-up only.

### Comparison of the Effect of Operative and Non-Operative Treatment on Functional Scores

Analysis of functional scores from two studies[15,16] showed statistically-significant differences favoring operative treatment at 2 and 3 months, however, at 6 and 12 months, the difference was not significant. Pooled data from the two studies reporting outcomes after more than 1y also demonstrated no significant difference (Table 2).

**Table 2:** Comparison of operative and non-operative treatments with respect to the outcomes of functional scores, range of motion, grip and pinch strength.

Results	Time	Studies	Fractures (O)	Fractures (N)	WMD/SMD	95% CI	*P* value	Favors
Function score	2 months	2	73	73	-0.77	-1.11∼-0.43	< 0.001	O
	3 months	2	71	69	-0.63	-1.16∼-0.11	0.02	O
	6 months	2	66	72	-0.42	-1.04∼0.21	0.19	
	1y	2	67	69	-0.19	-0.53∼0.15	0.26	
	>1y	2	70	67	-0.49	-3.84∼2.87	0.78	
Range of motion	2 months	2	73	73	25.01	-1.39∼51.38	0.06	
	3 months	2	71	69	12.55	-12.09∼37.18	0.32	
	6 months	2	66	72	3.83	-4.31∼11.96	0.36	
	1y	2	67	69	1.94	-2.75∼6.61	0.42	
	>1y	3	69	75	3.95	-2.17∼10.07	0.21	
Grip Strength	2 months	2	73	73	23.60	12.53∼34.68	< 0.001	O
	3 months	2	71	69	14.39	1.380∼27.40	0.03	O
	6 months	2	66	72	8.80	2.43∼15.17	0.007	O
	1y	2	67	69	7.40	1.52∼13.28	0.01	O
	>1y	3	86	84	4.38	-4.14∼12.31	0.33	
Pinch strength	>1y	2	75	71	-0.38	-5.31∼4.57	0.88	

^¶^, SMD data, O, operative treatment; N, non-operative treatment.

### Comparison of Operative and Non-Operative Treatment on Range of Motion, Grip and Pinch Strength

Of the six studies, three contained continuous data on ROM. No ROM variables revealed any significant differences in treatment effect at 2, 3, 6, or 12 months or more than 1y (Table 2).

Pooled data across two or three studies at 2, 3, 6, and 12 months’ follow-up demonstrated that those patients who underwent operative treatment had significantly greater grip strength than those who received non-operative treatment. (Table 2). However, at a follow-up time of more than 1y, the difference was not significant (Table 2). Two studies [14,20] which reported pinch strength data at more than 1y revealed that the outcomes of both operative and non-operative treatments evaluated in this study were similar (Table 2).

### Comparison of Operative and Non-Operative Treatment on Duration of Absence from Work

**Fig 4:**
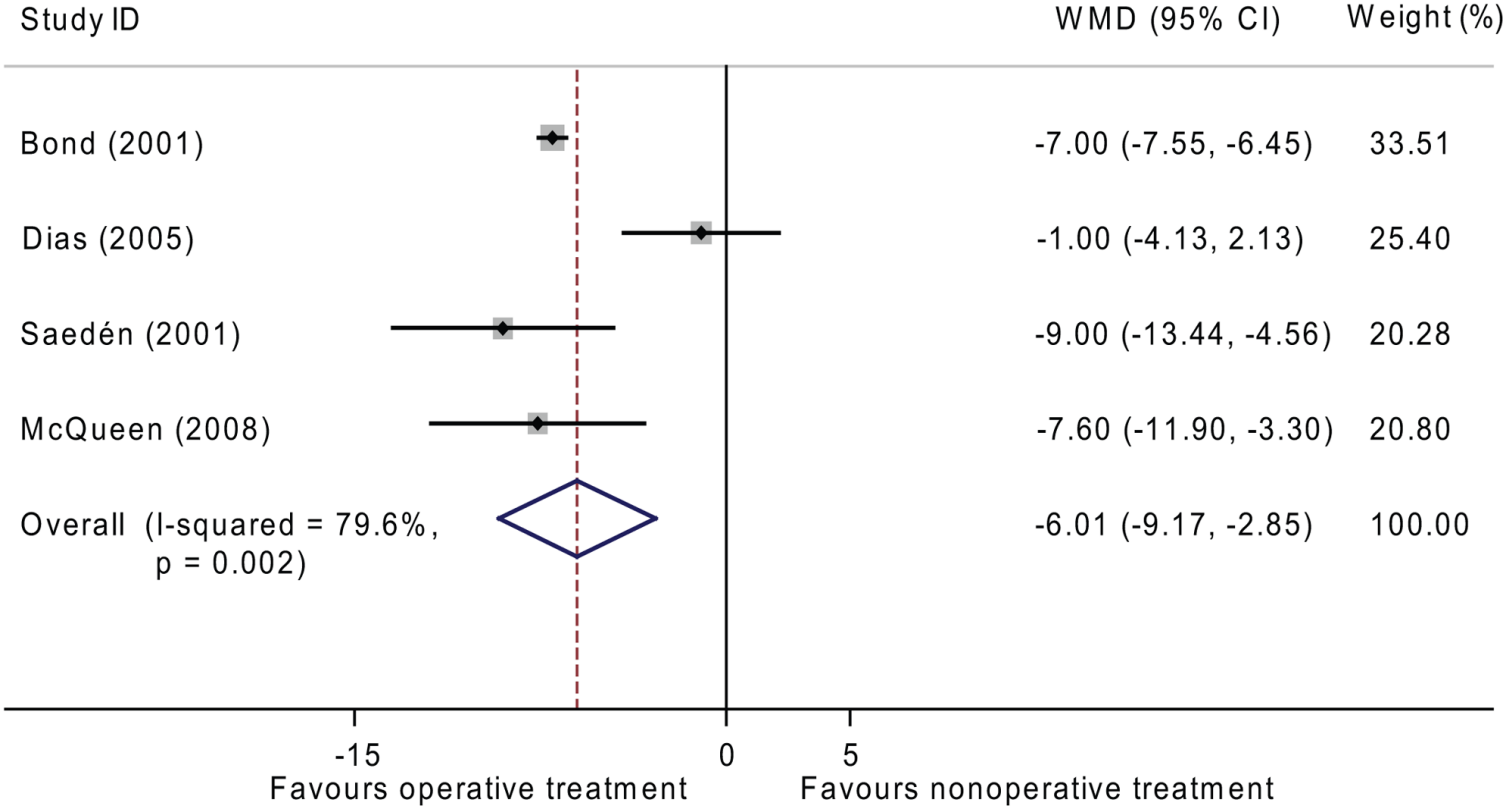
Forest plot for the WMD estimate for duration of absence from work. ■, weighting given to the trial in the overall pooled estimate, taking into account the number of participants and the amount of inter-study variation (heterogeneity); rhombus, combined effect size.

The duration of absence from work was reported in four of the six studies. Meta-analysis showed that patients who received operative treatment had the advantage regarding duration of absence from work as compared to those who underwent non-operative treatment (WMD = -6.01, 95% CI: -9.17 to -2.85, *P* < 0.001) (Fig 4).

### Comparison of Operative and Non-Operative Treatment on the Incidence of Complications and Time to Fracture Union

**Fig 5:**
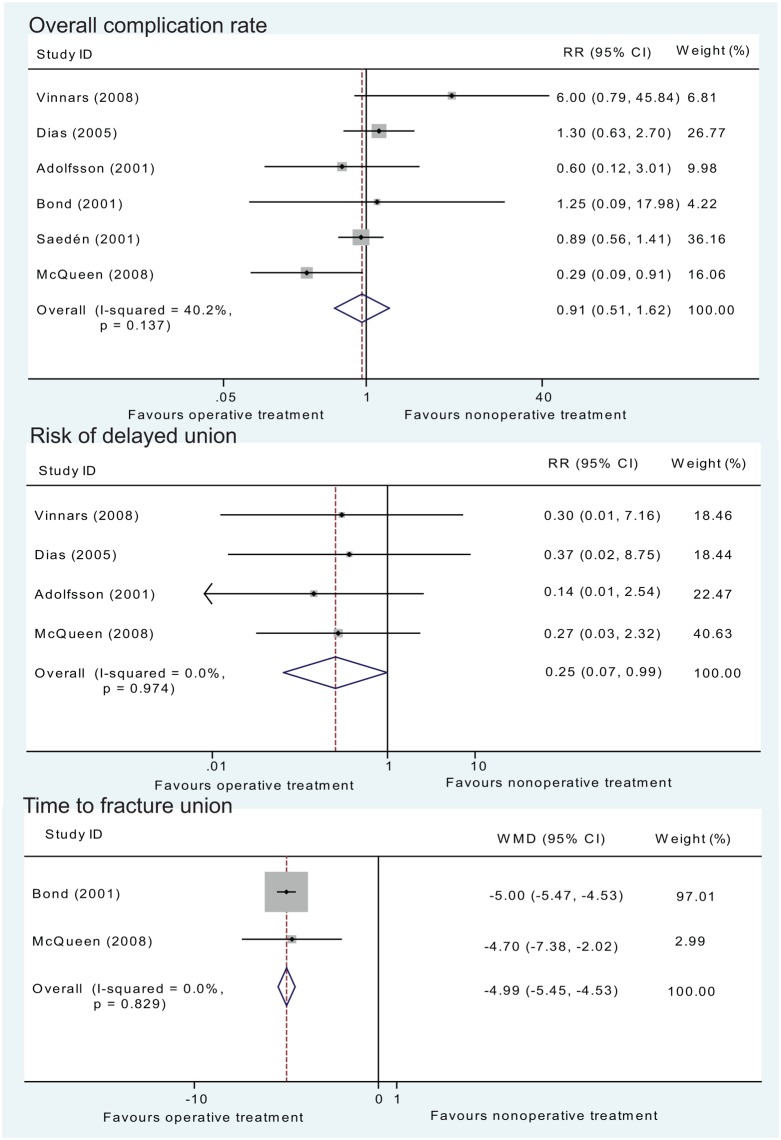
Assessment of the complication rate and time to fracture union of operative versus non-operative treatments. Upper graph, forest plot for the risk ratio (RR) estimate for overall complication rate. Middle graph, independent analysis regarding the risk of delayed union. Bottom graph, forest plot for the WMD estimate for time to fracture union. ■, weighting given to the trial in the overall pooled estimate, taking into account the number of participants and the amount of inter-study variation (heterogeneity); rhombus, combined effect size.

Regarding the overall complication rate, there was no statistically significant difference between operative and non-operative treatment (RR = 0.91, 95% CI: 0.51–1.62, *P* = 0.75) in six studies (Fig 5).

Independent analysis based on specific complications showed that the risk of delayed union was significantly lower with operative treatment (*P* = 0.049), with a 74.6% reduction (RR = 0.25, 95% CI: 0.07–0.99) in risk when compared with non-operative treatment (Fig 5). Number-needed-to-treat analysis demonstrated that if all patients underwent screw fixation, approximately 23 (22.72) patients would need to undergo surgery to prevent one delayed union. To further explore this result, an analysis regarding time to fracture union was performed and revealed a statistically-significantly difference, showing that the time was shorter with operative treatment (WMD = -4.99, 95% CI: -5.46 to -4.53, *P* < 0.001) (Fig 5). Meta-analysis regarding nonunion, further surgery, malunion, osteoarthritis and symptomatic osteoarthritis revealed no significant differences between operative and non-operative treatments (Table 3).

**Table 3:** Effect of operative versus non-operative treatments on the occurrence of complications.

Complications	Studies	Fractures (O)	Fractures (N)	RR	95%CI	P value
**Nonunion**	3	86	87	0.41	0.05–3.26	0.40
**Malunion**	2	69	72	0.22	0.03–1.99	0.18
**Symptomatic osteoarthritis**	2	54	49	0.70	0.37–1.35	0.29
**Osteoarthritis**	4	121	112	1.14	0.61–2.12	0.69
**Further surgery intervention**	5	145	147	1.52	0.36–6.08	0.55

O, operative treatment; N, non-operative treatment

### Sensitivity Analysis and Publication Bias Analysis

**Fig 6:**
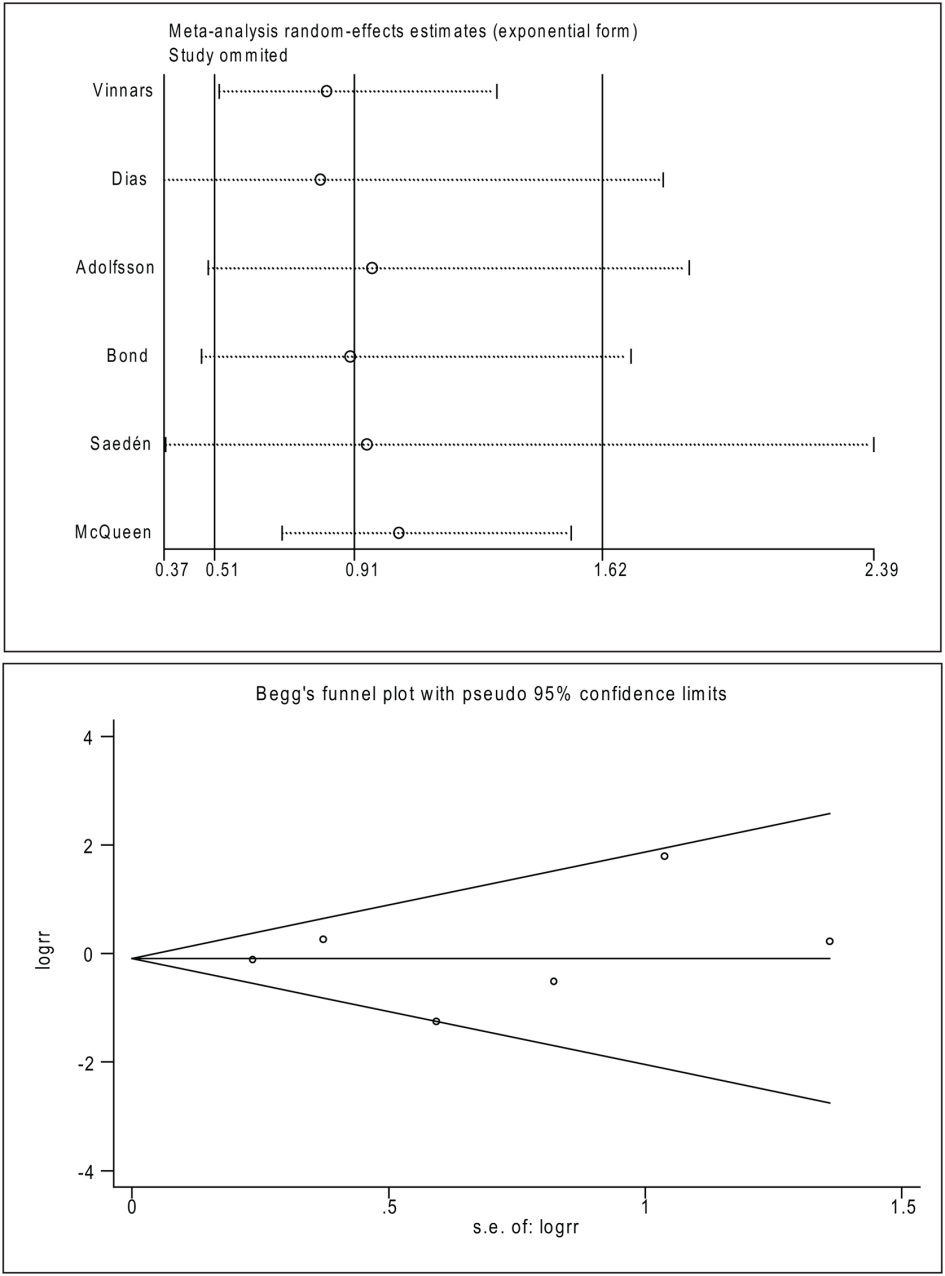
Sensitivity analysis and publication bias analysis of the meta-analysis. Upper graph: the influence of individual studies on the summary RR were computed by omitting each study in turn. The vertical axis indicates the overall RR and the two vertical axes indicate its 95% CI. Every circle indicates the pooled RR when the left study is omitted in this meta-analysis. The two ends of the dotted lines represent the 95% CI. Bottom graph: Begger’s funnel plot of publication bias in the selection of studies. The vertical axis represents the log [RR] and the horizontal axis indicates the standard error of the log [RR]. The horizontal line and the sloping lines represent the effects summary RR and the expected 95% CI for a given standard error, respectively. Each circle represents an independent study.

Sensitivity analysis and publication bias analysis was performed for the overall populations using the rate of incidence of overall complications. The evaluation regarding the influence of each study on the overall RR revealed that no individual study had a dominant effect on the overall RR, since omission of any single study did not make a large difference (Fig 6). Moreover, no significant publication bias was shown for the overall populations by the funnel plot (*P* = 0.71, Fig 6).

## Discussion

There was a wide range of follow-up term and a great deal of variation in the outcome measurements used across the included RCTs, with three of the six studies regarded as notably poor quality[17,18,19]. This variability suggests that there is a need for a better standardized protocol in any future study to accurately compare operative versus non-operative treatment.

Our results revealed that surgical treatment leads to a significantly better functional score in the early stages (2 and 3 months) after fracture fixation. One possible explanation is that screw fixation is more reliable and therefore achieves adequate stability and allows early wrist mobilization, leading to improved strength. This theory is supported by the grip strength outcomes in our analysis, with the operative treatment group showing significantly better grip strength than the non-operative treatment group from 2 months after fixation, an advantage which continued up to 12 months. Since better functional outcomes can be achieved in the short-term with screw fixation, operative treatment should be considered for patients who require a fast restoration of function. Consistent with these explanations, as shown in our study, patients treated surgically had a more rapid return to work compared with those managed non-surgically, based on the data regarding duration of absence from work.

Our meta-analysis demonstrated no statistically-significant difference between operative and non-operative treatment with regard to total complications. However, independent analysis showed that operative treatment had the advantage with regard to delayed union, which is supported by the analysis based on time to fracture union. Our findings contribute to the growing body of evidence that primary surgical treatment is a reliable method of reducing the risk of delayed union after acute undisplaced or minimally-displaced scaphoid fractures. However, a number-needed-to-treat analysis revealed that more than 20 patients would have to undergo operative treatment to prevent a single delayed union. Routine screw fixation would therefore expose an unacceptably high number of patients to the risks of surgery, especially when another meta-analysis[10] as well as the data of our study have shown that the risk of non-union is not significantly different between operative and non-operative treatments. The results of our analysis regarding complications differ from those of previous meta-analyses[8,10], which analyzed data including an ineligible study and others with flawed methods[8], or only performed the pooled analysis on the overall rate of complications, fracture union rate, and osteoarthritis of the scaphotrapeziotrapezoid joint between treatments[10]. With exclusion of the ineligible study and correction for improper data, and a pooled analysis of all available data, we believe that our results are more precise than other previously-published meta-analyses, supported by the sensitivity analysis and publication bias analysis, which showed that our result is stable and showed no publication bias.

Our meta-analysis has some limitations. The first limitation is that the sample size of each included study is small because of the difficulties of performing clinical trials in surgery, and given the small difference in outcomes such as nonunion and malunion, even the results from our pooled analysis are probably underpowered. The second limitation is that the effects of age on the outcomes of operative and non-operative treatment were not estimated in our analysis, due to the lack of data regarding this aspect in the included RCTs. In contrast to classical teaching, scaphoid fractures do occur even in the elderly population[21], and elderly patients tend to have lower demands of wrist function compared with younger patients. In addition, it was reported that the preoperative fracture pattern is significantly related to fracture nonunion and delayed union[22], but our meta-analysis did not reveal any effects of fracture-type-specific factors on the outcomes when operative and non-operative treatments were compared because no data of the included studies were available. Finally, whether the clinical benefits of surgery justify the financial costs associated with operative treatment remains unclear. A study by Arora *et al*.[9] revealed that the mean primary costs (radiographs, medical visits, plaster and plaster replacement, surgery) were significantly higher and the secondary costs (work compensation cost, therapy costs) were significantly lower in surgically-treated patients than in non-surgically-treated patients. Overall, operative treatment was less expensive than conservative treatment, but the difference was not significant. Vinnars *et al*.[23] compared the direct and indirect costs of internal fixation and cast treatment in acute scaphoid fractures based on one of the included studies[14] and revealed that in non-manuals, total costs were lower after casting than after surgery. However, the financial costs were not calculated in the other RCTs included in our meta-analysis, so we were unable to perform an analysis regarding this aspect.

Despite the limitations, we believe that this meta-analysis offers useful conclusions based on the published RCTs. In the management of acute undisplaced or minimally-displaced scaphoid fractures, patients who were treated surgically recovered more quickly than did patients who were treated non-surgically. Our data also indicate that the literature does not provide evidence supporting the routine use of operative treatment for all acute undisplaced or minimally-displaced scaphoid fractures.

In future clinical studies, researchers should design the protocol carefully, compare the effects of operative versus non-operative treatment in large multicenter trials that include both young and old patients with different types of fractures, and perform parallel economic analysis to obtain robust and comprehensive results.

## Supporting Information

S1 File PRISMA checklist of the Meta-analysis. (DOC)

S2 File Search equations used for all the databases. (DOC)
