# Supplementary material for: Comparison of Operative and Non-Operative Treatment of Acute Undisplaced or Minimally-Displaced Scaphoid Fractures: A Meta-Analysis of Randomized Controlled Trials
Source: PLoS One. 2015 May 5;10(5):e0125247. doi: 10.1371/journal.pone.0125247 (PMC4420279; doi:10.1371/journal.pone.0125247)
Supplement: S2 File — (DOC) [file pone.0125247.s002.doc]

Search equations used for all the databases

PubMed

(("scaphoid bone"[MeSH Terms] OR ("scaphoid"[All Fields] AND "bone"[All Fields]) OR "scaphoid bone"[All Fields] OR "scaphoid"[All Fields]) AND ("fractures, bone"[MeSH Terms] OR ("fractures"[All Fields] AND "bone"[All Fields]) OR "bone fractures"[All Fields] OR "fractures"[All Fields])) AND "randomized controlled trial"[Publication Type]

CENTRAL

search on 'scaphoid fractures in Title, Abstract, Keywords in Trials'

BioMed Central

scaphoid fractures (All words) in All fields (full text)

ScienceDirect

Scaphoid fracture in Abstract, Title, Keywords AND randomized controlled trials in Full Text

Web of Science

TS= scaphoid AND TI= randomized
